# Zika virus epidemiology in Bolivia: A seroprevalence study in volunteer blood donors

**DOI:** 10.1371/journal.pntd.0006239

**Published:** 2018-03-07

**Authors:** Paola Mariela Saba Villarroel, Elif Nurtop, Boris Pastorino, Yelin Roca, Jan Felix Drexler, Pierre Gallian, Thomas Jaenisch, Isabelle Leparc-Goffart, Stéphane Priet, Laetitia Ninove, Xavier de Lamballerie

**Affiliations:** 1 UMR EPV Émergence des Pathologies Virales, Aix-Marseille University—IRD 190—Inserm 1207 –EHESP–IHU Méditerranée Infection, Marseille, France; 2 Virología II, Centro Nacional de Enfermedades Tropicales (CENETROP), Santa Cruz de la Sierra, Bolivia; 3 German Centre for Infection Research (DZIF) Charité—Universitätsmedizin Berlin, Berlin, Germany; 4 Institute of Virology, Charité—Universitätsmedizin Berlin, Berlin, Germany; 5 Laboratoire de Virologie, Établissement Français du Sang Alpes Méditerranée (EFS), Marseille, France; 6 Department for Infectious Diseases (Section Clinical Tropical Medicine), Heidelberg University Hospital, Heidelberg, Germany; 7 National Reference Centre for Arboviruses, French Armed Forces Biomedical Research Institute, Marseille, France; University of California San Francisco, UNITED STATES

## Abstract

**Background:**

Zika virus (ZIKV), was widely reported in Latin America and has been associated with neuropathologies, as microcephaly, but only few seroprevalence studies have been published to date. Our objective was to determine the seroprevalence amongst Bolivian blood donors and estimate the future potential circulation of the virus.

**Methodology:**

A ZIKV seroprevalence study was conducted between December 2016 and April 2017 in 814 asymptomatic Bolivian volunteer blood donors residing in various eco-environments corresponding to contrasting entomological activities. It was based on detection of IgG to ZIKV using NS1 ELISA screening, followed by a seroneutralisation test in case of positive or equivocal ELISA result.

**Conclusions/Significance:**

Analysis revealed that ZIKV circulation occurred in tropical areas (Beni: 39%; Santa Cruz de la Sierra: 21.5%) but not in highlands (~0% in Cochabamba, La Paz, Tarija). It was modulated by *Aedes aegypti* activity and the virus spread was not limited by previous immunity to dengue. Cases were geo-localised in a wide range of urban areas in Santa Cruz and Trinidad. No differences in seroprevalence related to gender or age-groups could be identified. It is concluded that ZIKV has been intensely circulating in the Beni region and has still a significant potential for propagating in the area of Santa Cruz.

## Author summary

Zika virus (ZIKV) is a virus of African origin, transmitted by *Aedes* mosquitoes, and related to dengue and yellow fever virus. It was originally believed to be responsible for a mild febrile illness in Africa and South-east Asia. However, in recent years, ZIKV has been responsible for outbreaks in the Pacific Islands before massively spreading in Latin America and the Caribbean. On this occasion, ZIKV has unexpectedly been associated with non-vector transmission (*i*.*e*., sexual and mother-to-foetus transmission) and with severe complications such as foetal abnormalities *(e*.*g*. microcephaly) and Guillain-Barré syndromes.

Little is known about the actual proportion of the populations infected by ZIKV in Latin America. Here, we report a seroprevalence data in this region, after studying 814 asymptomatic Bolivian volunteer blood donors residing in various eco-environments corresponding to contrasting entomological activities. We conclude that ZIKV has been circulating in Bolivian tropical areas but not in highlands, and that the epidemic has not been limited by previous immunity against dengue. Specific attention should be paid to the region of Santa Cruz, where the seroprevalence is still limited, but the density of *Aedes aegypti* populations makes plausible further spreading of the disease.

## Introduction

Zika virus (ZIKV) is an arthropod-borne *Flavivirus* transmitted to humans mainly by *Aedes* mosquitoes. It has been responsible over the last decade for outbreaks in Pacific Islands [1] (Yap Island, 2007 [2]; French Polynesia, 2013 [3]; New Caledonia, Cook Islands and Easter Island, 2014 [4]; Vanuatu, Solomon Islands, Samoa and Fiji, 2015 [5]), in Latin America (from late 2013 [6] or early 2014 [7] in Brazil, then in a large number of other countries [8]), and in the Caribbean region (*e*.*g*., 2014 in Haiti [9], 2015 in Martinique Island [10]). The first autochthonous case reported in Bolivia was in January 2016 [11].

ZIKV is of African origin, but an Asian lineage emerged presumably in the first part of the XIX^th^ century [6] and the viruses that spread in the Pacific and the Americas are descendants of this lineage. In addition to the classical picture of large arboviral outbreaks, significant public health burden was endured in Polynesia and America when severe and formerly undescribed foetal and neurological complications of the disease [12] as well as non-vectored routes of transmission [13] were reported.

Our capacity to estimate the future spread of ZIKV disease in South America significantly depends on our knowledge of the immune status against ZIKV in the populations exposed to the potential vectors (mostly *Aedes aegypti* mosquitoes). Few ZIKV seroprevalence results in the region have been made available yet, most probably due to the technical difficulty to distinguish antibodies to ZIKV from cross-reacting antibodies to the other flaviviruses, in particular dengue virus. Specific detection of antibodies to ZIKV can be improved when using demanding seroneutralisation methods for the primary detection of antibodies, or for confirmation of a more convenient screening assay such as an ELISA test.

In this context, we investigated the ZIKV serological status of blood donors from different regions of Bolivia and analysed results with reference to immunity of the same populations to dengue (DENV) and chikungunya virus (CHIKV), two arboviruses known to be transmitted by the same vector locally. DENV has been reported in Bolivia since 1931. In 1948, Bolivia was declared *Ae*. *aegypti* free, but the vector reappeared in the 1980s. Since then, DENV-1, DENV-2, DENV-3, lately DENV-4 and the co-circulation of serotypes have been reported, being massively endemic in the tropical regions of Bolivia [14, 15]. CHIKV, a member of the *Alphavirus* genus arrived in the Caribbean in late 2013 and then spread through Latin America the following years, causing explosive outbreaks in humans[16].

## Methods

### Sample collection

We conducted a study with the help of five Bolivian regional blood banks, representing a variety of eco-environments (Santa Cruz de la Sierra and Beni have tropical climate; Cochabamba, Tarija and La Paz have colder subtropical highland climates): 814 volunteer blood donors from Santa Cruz de la Sierra (n = 200), La Paz (n = 161), Cochabamba (n = 152), Tarija (n = 196), and Beni (n = 105) provided before blood donation their oral consent for detection of IgG to ZIKV. All donors accepting to participate in the study and providing consent were considered eligible. No specific sampling was performed and leftovers of blood samples collected and stored at -20°C after completion of laboratory analyses were used. The contribution of each site was evaluated locally according to the actual possibilities to recruit donors, process samples and provide the epidemiological data requested (see below). The sample size was in agreement with previous epidemiological studies of arbovirus seroprevalence in other countries and expected to provide a reliable picture of the global epidemiological situation [17, 10]. Sampling was performed in December 2016 in the Beni region, then from March to April 2017 in the other sites, according to local logistical possibilities.

### Ethics statement

Blood samples and personal data (date of donation, sex, age, birthplace, living place, occupation and neighbourhood) were irreversibly anonymised.

Adults blood donors approved to participate in the study by providing oral consent during the face-to-face questioning before blood gift. This procedure was considered the most suitable by local blood banks. The sampling and analysis protocol was approved by the ethics committee of the Medical College of Santa Cruz.

### Serological analysis

Samples were tested for the presence of IgG to ZIKV as previously described [10,17]. In brief, alike Netto and collaborators[18] we performed an initial screening with a recombinant NS1-based ELISA test (Euroimmun, Lübeck, Germany) [19, 20] which is the only test certified for serological diagnostics of ZIKV by the responsible Brazilian authority ANVISA (Agência Nacional de Vigilância Sanitária)[18] and a subsequent Virus Neutralisation Test (VNT) for samples with a positive or equivocal ELISA result (ratio ≥0.8). VNT was performed in a 96-well format based on cytopathic effect (CPE), using ZIKV strain H/PF/2013[21], Vero ATCC cells monolayers and serum dilutions from 1:10 to 1:320. All samples were tested in duplicate with positive and negative serum controls. Sera with titre ≥1:40 were considered positive, according to the recommendations of the French National Reference Centre for Arboviruses. This testing strategy was previously demonstrated to provide specificity and sensitivity values above 98.5% in volunteer blood donors of Martinique Island tested before, during, and after the 2016 ZIKV outbreak[22] and with heavy exposure to dengue[23].

To estimate the serological immune background against dengue and chikungunya (which in Bolivia are also transmitted by *Aedes aegypti*), we randomly selected approximately half of the samples on each site (Beni, n = 60; Santa Cruz de la Sierra, n = 108; Tarija, n = 111; La Paz, n = 93; Cochabamba, n = 77; *i*.*e*., 449 in total). They were tested for the presence of IgG to DENV and CHIKV (Euroimmun dengue ELISA IgG and chikungunya ELISA IgG assays) according to the manufacturer's recommendations.

Donors were assigned for analysis to 3 age-groups (18–30 years-old; 31-40yo; 41-60yo) and 6 ZIKV ELISA ratio groups (<0.8; 0.8–1.09; 1.1–2.49; 2.5–3.99; 4.0–5.49; ≥5.5).

Statistical analyses were performed using IBM-SPSS Statistics v 24.0.0.0 software. Statistical association between ZIKV seropositivity and age-group or gender was evaluated, as well as relationship between ZIKV seropositivity and DENV or CHIKV seropositivity (Chi square test, significant threshold: p = 0.05).

#### Accession numbers

Zika virus strain H/PF/2013: KJ776791.2

## Results and discussion

**Fig 1:**
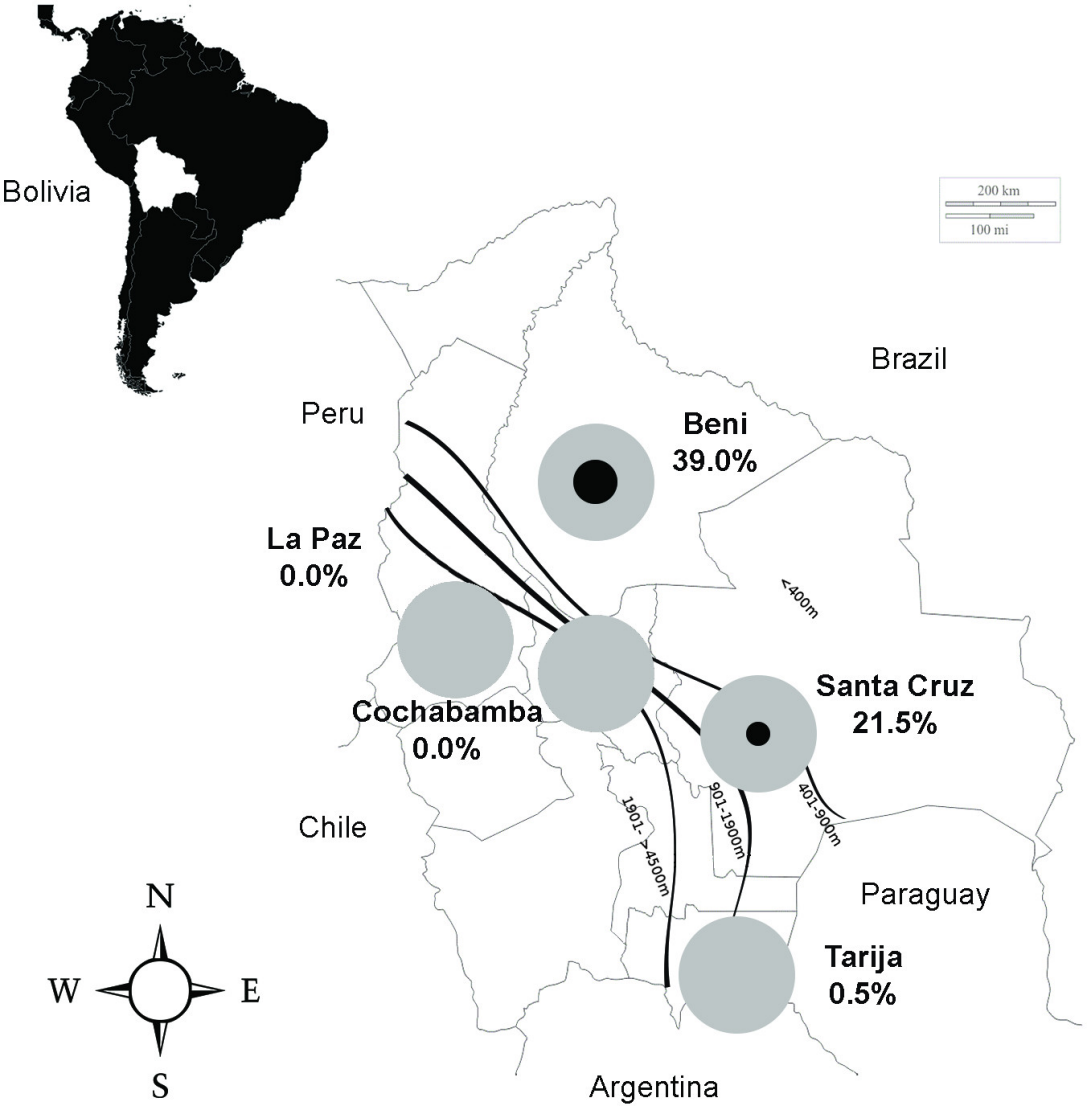
Zika virus seroprevalence in Bolivian volunteer blood donors. Samples were collected in December 2016 (Beni) and from March to April 2017 (other regions). The figure shows in each of the five regions investigated the proportion of samples positives in both ELISA and Virus Neutralisation Test (the black circle represents the proportion of positives): Santa Cruz (altitude ~400m) and Beni (alt. ~150m) have tropical climate; Cochabamba (alt. ~2500m), Tarija (alt. ~1850m), and especially La Paz (alt. ~3650m) have colder subtropical highland climates.

Serological results in the different sites for ZIKV are shown in Fig 1. ZIKV has been circulating in the two regions with a typical tropical climate (Beni and Santa Cruz de la Sierra), but not in highlands in which the entomological activity is limited. In the tropical regions, the usual period of circulation of *Aedes* borne viruses ranges from November to April. The high seroprevalence rate in Beni (39% after VNT, (95% CI [30%–48%])) is in agreement with the report of an outbreak of febrile cases with rash locally, with laboratory PCR confirmed cases before our study (performed in December 2016). Since cases were reported in Beni until the end of the rainy season, it is likely that the final seroprevalence rate is even higher nowadays in this region.

The significant rate in Santa Cruz (21.5% after VNT, (95% CI [16%-27%])) is in line with the report of PCR confirmed cases locally before the collection of samples (in March and April 2017). Detection of cases benefitted from the local implementation of the Cenetrop National Reference Laboratory, but no clear epidemic pattern was reported. Since the study was performed locally at the end of the rainy season, it is most probable that the seroprevalence rate remained locally at a lower level than in Beni.

Of note, the proportion of ELISA positives confirmed by VNT was much higher in Beni than in Santa Cruz (63.1% *vs* 35.2%), possibly reflecting the frequent and intense immune stimulation against ZIKV in the Beni population.

When examining the relationship between ZIKV ELISA and VNT results, it appears, as previously observed [10, 17], that the rate of VNT confirmation increases with the value of the ELISA ratio, reaching 65% for an ELISA ratio ≥4 and 95% for an ELISA ratio ≥5.5 (S1 Table).

Table 1 shows the distribution of seropositives per site with the background of serological results for dengue and chikungunya, which circulation in Bolivia has been widely documented. Immunity to dengue virus is the rule in the tropical regions (>90% of seropositives in Beni and Santa Cruz), but also significant in the region of Tarija (44.1%). It is more limited in Cochabamba and La Paz (*ca* ~10%). For chikungunya, approximately half of the population is seropositive in the tropical regions and less than 10% in highlands. The differences observed with dengue most probably reflect the lower number of epidemic waves of chikungunya that hit the country. Dengue was first reported in Bolivia in 1931 and since then has been circulating intensely with major epidemics in the tropical regions[15] and, over time, multiple imported cases and possibly transient local transmission in highlands (in particular the Tarija region). By contrast, autochthonous transmission of CHIKV was first reported in Bolivia as recently as 2015 following the introduction of the virus in the Americas[24], limiting herd immunity outside the areas of intense epidemic transmission. Altogether, the spreading pattern of ZIKV follows that of other *Aedes aegypti*-borne viruses in Bolivia, in particular that of the recently introduced CHIKV.

**Table 1:** Serological results for Zika, dengue and chikungunya in Bolivian blood donors.

IgG ELISA	Total No.	Positive No. (% [95% CI])	Equivocal No. (%)	Negative No. (%)	VNT^a^	No.	Positive No. (% [95% CI])	VNT/ELISA (%)
**Zika**					**Zika**			
**Beni**	105	65 (61.9)	5 (4.8)	35 (33.3)		70	41 (39.0 [30–48])	63.1
**Santa Cruz**	200	122 (61.0)	12 (6.0)	66 (33.0)		134	43 (21.5 [16–27])	35.2
**Tarija**	196	19 (9.7)	3 (1.5)	174 (88.8)		22	1 (0.5 0–1.5])	5.3
**La Paz**	162	3 (1.9)	1 (0.6)	157 (96.9)		4	0 (0.0)	0.0
**Cochabamba**	152	4 (2.6)	3 (2.0)	145 (95.4)		7	0 (0.0)	0.0
**Chikungunya**				
**Beni**	60	28 (46.7 [34–59])	0 (0.0)	32 (53.3)
**Santa Cruz**	108	59 (54.6 [45–64])	1 (0.9)	48 (44.4)
**Tarija**	111	6 (5.4 1–10])	0 (0.0)	105 (94.6)
**La Paz**	93	3 (3.2 0–7])	0 (0.0)	90 (96.8)
**Cochabamba**	77	6 (7.8 2–14])	0 (0.0)	71 (92.2)
**Dengue**				
**Beni**	60	54 (90.0 [82–98])	0 (0.0)	6 (10.0)
**Santa Cruz**	108	101 (93.5 [89–98])	1 (0.9)	6 (5.6)
**Tarija**	111	49 (44.1 [35–53])	3 (2.7)	59 (53.2)
**La Paz**	93	11 (11.8 5–18])	0 (0.0)	82 (88.2)
**Cochabamba**	77	8 (10.4 4–17])	0 (0.0)	69 (89.6)

Serology for Zika virus was performed for the complete population of the study using a commercial NS1-based IgG ELISA screening assay followed by a Virus Neutralisation Test (VNT) for samples with ELISA positive or equivocal results. Serology for dengue and chikungunya viruses was performed for a randomly selected sample corresponding to *ca*. fifty per cent of the complete population studied. Testing was performed with commercial assays using purified virus particles (dengue) and recombinant proteins (chikungunya) as viral antigens. Percentages are expressed as the proportion of positives in the total population tested in ELISA.

^**a**^Virus Neutralisation test

S2 Table details the distribution of seropositives for ZIKV, DENV and CHIKV according to sex and age groups. Differences are minimal between groups (none reaches a significance threshold of 0.05), suggesting that age and sex do not significantly impact exposure to these arboviral diseases in the population investigated. Importantly, there is in the population studied a strong relationship between seropositivity to ZIKV and to DENV (p<10^−12^), but also to CHIKV (p<10^−15^), obviously pointing to exposure to a common risk factor: the bite of the *Aedes aegypti* vector of the three diseases.

This study has classical limitations linked to the sampling procedure in blood donors (in particular data regarding individuals under the age of 15 years old and in pregnant women could not be obtained). However, many previous studies on arboviruses including dengue virus[23] chikungunya virus[25], Zika virus [10] have suggested that blood donors constitute a valuable population to identify the major epidemiological trends that underlie exposure to arbovirus transmission and spread. Amongst great advantages of studying blood donor's populations, one can mention the logistical capability to perform rapidly multisite studies in the absence of robust local research infrastructure, the absence of the need to perform specific sampling, and the access to comparable populations in multiple sites that allows comparison of prevalence values.

We conclude that this seroprevalence study confirms the circulation of Zika virus in Bolivia. Despite previous reports of the presence of *A*. *aegypti* in all 5 departments investigated [14, 26, 27, 28, 29], the potential for epidemic spread is deeply modulated by the variable entomological activity in the different locations, in relation with different eco-environments (and in particular different altitudes) and as reflected by contrasted exposure to dengue or chikungunya. In the tropical areas of Santa Cruz and Beni, cases were identified from the city centres to outlying district, reflecting the wide distribution of *A*. *aegypti*. According to previous information relating to the circulation of dengue in Bolivia and corroborated by our present seroprevalence data, the spread of Zika virus was not limited by previous herd immunity to dengue virus. However, it remains possible that prior immunity to DENV modified the epidemiological pattern of the virus global spread.

The same methodological protocol has been used previously to estimate ZIKV seroprevalence in Martinique Island [10] (Caribbean region) and Cameroon[17] (Central Africa). Clearly, the transmission pattern in Bolivia is very different from the (peri-)sylvatic transmission reported in Cameroon, and more closely related to the urban transmission by the (peri-)domestic *A*. *aegypti* in Martinique. With reference to the Martinique outbreak and seroprevalence study, a minimum seroprevalence rate around 50% seems to be required to provide herd immunity that can stop ZIKV circulation. Accordingly, the ~21% rate observed in Santa Cruz (in the last phase of the arbovirus circulation period) would be insufficient to give protective herd immunity in the presence of abundant potential vectors and intense entomological activity, and with sustained circulation and potential reintroduction of the virus in Latin America. It is therefore expected that ZIKV circulation should be limited in the near future in the Beni Region (data were collected in December and the virus could circulate for at least four additional months locally, therefore the final seroprevalence rate may be even higher), but, in contrast, ecological and epidemiological conditions are favourable for further circulation of the virus in Santa Cruz, with its consequent complications especially in pregnant women.

## Supporting information

S1 Checklist STROBE checklist. (PDF)

S1 Table Relation between ELISA ratio and Virus Neutralisation Test. (DOCX)

S2 Table Seropositivity for Zika, dengue and chikungunya virus, according to sex and age group. Prevalence between males and females were compared for each site and globally using the chi-square test and were insignificant for p-value at 0.05.
(DOCX)
