# Supplementary material for: Zika virus epidemiology in Bolivia: A seroprevalence study in volunteer blood donors
Source: PLoS Negl Trop Dis. 2018 Mar 7;12(3):e0006239. doi: 10.1371/journal.pntd.0006239 (PMC5858838; doi:10.1371/journal.pntd.0006239)
Supplement: S1 Table — (DOCX) [file pntd.0006239.s002.docx]

|  |  | **VNT* positives** | | | | |
| --- | --- | --- | --- | --- | --- | --- |
| **Zika** | negatives  ELISA ratio:  <0.8  No. (%) | equivocals  ELISA ratio:  0.8-1.09  No. (%) | positives  ELISA ratio:  1.1-2.49  No. (% [95% CI]) | positives  ELISA ratio:  2.5-3.99  No. (% [95% CI]) | positives  ELISA ratio:  4.0-5.49  No. (% [95% CI]) | positives  ELISA ratio:  ≥5.5  No. (% [95% CI]) |
| Beni | 0 (0.0) | 0 (0.0) | 3 (20.0 [0-40]) | 7 (63.6 [35-92]) | 15 (71.4 [52-91]) | 16 ( 94.0 [83-100]) |
| Santa Cruz | 0 (0.0) | 0 (0.0) | 8 (14.5 [5-24]) | 12 (34.3 [19-50]) | 17 (65.4 [47-84]) | 6 (100.0) |
| Tarija | 0 (0.0) | 0 (0.0) | 0 (0.0) | 1 (16.7 [0-47]) | 0 (0.0) | 0 (0.0) |
| La Paz | 0 (0.0) | 0 (0.0) | 0 (0.0) | 0 (0.0) | 0 (0.0) | 0 (0.0) |
| Cochabamba | 0 (0.0) | 0 (0.0) | 0 (0.0) | 0 (0.0) | 0 (0.0) | 0 (0.0) |

*VNT: Virus neutralisation test
