# Supplementary material for: Zika virus epidemiology in Bolivia: A seroprevalence study in volunteer blood donors
Source: PLoS Negl Trop Dis. 2018 Mar 7;12(3):e0006239. doi: 10.1371/journal.pntd.0006239 (PMC5858838; doi:10.1371/journal.pntd.0006239)
Supplement: S2 Table — Prevalence between males and females were compared for each site and globally using the chi-square test and were insignificant for p-value at 0.05. (DOCX) [file pntd.0006239.s003.docx]

|  | M | | F | |  | | 18-30yo | | 31-40yo | | >40yo | | |
| --- | --- | --- | --- | --- | --- | --- | --- | --- | --- | --- | --- | --- | --- |
|  | Total  No. | +ves  No. (% [95% CI]) | +ves  No. (% [95% CI]) |  | | Total  No. | | +ves  No. (% [95% CI]) | Total No. | +ves  No. (% [95% CI]) | Total No. | +ves  No. (% [95% CI]) |  |
| ZIKV (ELISA+VNT*) |  |  |  |  | |  | |  |  |  |  |  |  |
| Beni | 76 | 28 (36.8 [26-48]) | 13 (44.8 [27-63]) |  | | 46 | | 21 (45.7 [31-60]) | 36 | 12 (33.3 [18-49]) | 23 | 8 (34.8 [15-54]) |  |
| Santa Cruz | 123 | 26 (21.2 [14-28]) | 17 (22.1 [13-31]) |  | | 112 | | 23 (20.5 [13-28]) | 63 | 16 (25.4 [15-36]) | 25 | 4 (16.0 [2-30]) |  |
| Tarija | 121 | 1 (0.8 [0 -2.4]) | 0 (0.0) |  | | 146 | | 1 (0.7 [0- 2]) | 34 | 0 (0.0) | 16 | 0 (0.0) |  |
| La Paz | 103 | 0 (0.0) | 0 (0.0) |  | | 96 | | 0 (0.0) | 39 | 0 (0.0) | 26 | 0 (0.0) |  |
| Cochabamba | 47 | 0 (0.0) | 0 (0.0) |  | | 92 | | 0 (0.0) | 36 | 0 (0.0) | 24 | 0 (0.0) |  |
| CHIKV (ELISA) |  |  |  |  | |  | |  |  |  |  |  |  |
| Beni | 50 | 25 (50.0 [36-64]) | 3 (30.0 [2-58]) |  | | 30 | | 14 (46.7 [29-65]) | 20 | 10 (50.0 [28-72]) | 10 | 4 (40.0 [10-70]) |  |
| Santa Cruz | 67 | 35 (52.2 [40-64]) | 24 (58.5 [43-74]) |  | | 66 | | 35 (53.0 [41-65]) | 27 | 18 (66.7 [49-84]) | 15 | 6 (40.0 [15-65]) |  |
| Tarija | 70 | 4 (5.7 [0-11]) | 2 (4.9 [0-12]) |  | | 86 | | 5 (5.8 [1-11]) | 15 | 0 (0.0) | 10 | 1 (10.0 [0-29]) |  |
| La Paz | 63 | 2 (3.2 [0- 8]) | 1 (3.3 [0-10]) |  | | 57 | | 1 (1.8 [0- 5]) | 20 | 2 (10.0 [0 -23]) | 16 | 0 (0.0) |  |
| Cochabamba | 23 | 1 (4.3 [0-13]) | 5 (9.3 [2-17]) |  | | 46 | | 4 (8.7 [1-17]) | 18 | 0 (0.0) | 13 | 2 (15.4 [0-35]) |  |
| DENV (ELISA) |  |  |  |  | |  | |  |  |  |  |  |  |
| Beni | 50 | 46 (92.0 [84- 99]) | 8 (80.0 [55-100]) |  | | 30 | | 26 (86.7 [74-99]) | 20 | 19 (95.0 [85-104]) | 10 | 9 (90.0 [71-100]) |  |
| Santa Cruz | 67 | 64 (95.5 [91-100]) | 37 (90.2 [81- 99]) |  | | 66 | | 61 (92.4 [86-98]) | 27 | 26 (96.3 [89-100]) | 15 | 14 (93.3 [81-100]) |  |
| Tarija | 70 | 28 (40.0 [29- 51]) | 21 (51.2 [36- 66]) |  | | 86 | | 36 (41.9 [31-52]) | 15 | 8 (53.3 [28- 79]) | 10 | 5 (50.0 [19- 81]) |  |
| La Paz | 63 | 9 (14.3 [6- 23]) | 2 (6.7 [0- 16]) |  | | 57 | | 5 (8.8 [2-16]) | 20 | 3 (15.0 [0- 31]) | 16 | 3 (18.7 [0- 38]) |  |
| Cochabamba | 23 | 2 (8.7 [0- 20]) | 6 (11.1 [3- 19]) |  | | 46 | | 5 (10.9 [2-20]) | 18 | 2 (11.1 [0- 26]) | 13 | 1 (7.7 [0- 22]) |  |

*VNT= Virus Neutralisation Test; ZIKV= Zika virus; CHIKV= Chikungunya virus; DENV= Dengue virus; +ves= Positive
